# How Class Influences the Ethnic Identity of Chinese Immigrants in the UK: Citizenship, Work, and Solidarity

**DOI:** 10.1111/1468-4446.70078

**Published:** 2026-01-28

**Authors:** Zhaowei Yin

**Affiliations:** ^1^ University of Glasgow Glasgow UK

**Keywords:** boundaries, Bourdieu, capital, class, ethnic identity, migration

## Abstract

One of the current focal points of ethnicity research is the relationship between ethnic identity and social inequality. This paper examines how immigrants' understandings of ethnicity are influenced by class. Through life‐history interviews with 28 Chinese immigrants in the UK, I focus on the experiences and feelings of immigrants from different social classes crossing borders, as well as how these experiences influence their understanding of ethnicity and identity, which often involve ethnically salient situations and the ways they draw ethnic boundaries. By focussing on immigrants' citizenship acquisition and work strategy development, I show three ways that class affects ethnicity: the extent of barriers when crossing borders, the ability to use transnational capital, and the forms of solidarity. This work contributes to the study of the intersection between ethnicity and class, revealing the heterogeneity of Chinese immigrants in the UK from a class perspective.

## 1 Introduction

When I lived with them (refugees), they all unified to cook… But the food they cook was terrible…We cannot cook (not allowed for security reasons) … At that time (we) were forced to eat with the British… Many people suffered from stomach‐aches… I regret coming here, I hate the British. I feel that I do not belong (here).

Gwen describes her unpleasant experience of living in a refugee camp when she first arrived in the UK and her views on the ‘British people’. The moving words show the sufferings of a marginalized Chinese immigrant in transnational process, and how this profoundly affects her understanding of ethnic identity. This implies that if she had not encountered such obstacles and terrible treatment, she might have treated her ethnicity differently. A very different kind of experience is conceivable and accessible for those in a higher class. Because the resources they possess and mobilize are richer and more diverse (Wang et al. 2020), which usually means higher social respect and a better quality of life in their immigrant lives (Wingen et al. 2020). Therefore, it is reasonable to assume that the way these immigrants understand ethnicity may be related to the class they belong to.

An increasing number of studies have shown interest in the various barriers and opportunities encountered by migrants in transnational mobility, as well as the ways they navigate these challenges (Erel and Ryan 2019; Trinidad and Faas 2025). Recent research has focussed on how migrants mobilizing capital and adopting strategies in their transnational practices according to specific social structures, and how these process are shaped by their social status (Erel 2010; Erel and Ryan 2019). However, there appears to be less research examining how these approaches and strategies influence migrants' understanding of their own ethnic identity. This paper argues that exploring the relationship between migrants' capital mobilization ways and their ethnic identities contributes to a deeper understanding of the intersection between class and ethnicity. At the same time, how ethnicity is socially constructed (Brubaker 2004; Jenkins 2008) through power (Wimmer 2013), institutions (Brubaker 2006; Lewis and Embrick 2016; Stewart and Sanders 2024) and other factors around boundaries (Wimmer 2008; Lamont et al. 2015; Edgell et al. 2020) is one of the core concerns of ethnic studies, which often involves an examination of the intersection of ethnicity with other social categories (Garcia 2017).

Accordingly, the article, based on qualitative research with the Chinese community Scotland, explores how class influences ethnic identity by analysing structural obstacles faced by the migrants in the UK, how this contributes to their ethnic identity formation by creating an ‘us’ and ‘them’ mentality (Jenkins 2008; Wimmer 2013). More specifically, the paper addresses three interrelated objectives: (1) to examine how class influences migrants' experiences of exclusion and thus the salience of ethnic identity in everyday life; (2) to analyse how migrants in different social positions draw ethnic boundaries differently, generating internal heterogeneity within the Chinese community; and (3) to explore how these dynamics shape the formation and maintenance of ethnic solidarities. In doing so, the paper contributes to migration and boundary‐making theories by examining how different forms of capital are converted and re‐evaluated across contexts, and how such conversions shape the drawing, reinforcement or blurring of ethnic boundaries.

This paper also contributes to the exploration of the heterogeneity of Chinese immigrants in the UK. As an increasingly growing and diverse group, Chinese migrants in the UK deserve greater attention, particularly in terms of their ethnic identity, social integration, and class composition. In the late 20th century, waves of immigrants from Hong Kong, a new generation of mainland Chinese students, and illegal immigrants arrived, making the Chinese migrant community in the UK more diverse (see Song 2011; Luk 2009), and one of the fastest growing ethnic minorities (Lau‐Clayton 2011). With the passage of time, their residential areas have expanded from the initially designated regions to a wider urban scope, and their occupational distribution has also evolved from early employment in the catering and retail industries to a broader range of business sectors (McFarland 1991; Pacione 2005). However, this internal diversity is often obscured in policy and public discourse, where Chinese migrants are frequently portrayed as a homogeneous group (Lam et al. 2009). Therefore, more research is needed to uncover the heterogeneity of this group, especially the significant internal differences in employment and identity (ibid).

## 2 Understanding Ethnicity

As the view that ethnicity is socially constructed has become the mainstream discourse in ethnic studies (Wimmer 2013), current research increasingly emphasizes the fluidity and relationality of ethnicity (see Jenkins 2008; Brubaker 2004, 2009; Wimmer 2013, etc.). As a social classification that describes group relationships, ethnicity can best be understood by attending to the ‘boundaries’ and ‘interactions’ of groups (Eriksen 2010; Jenkins 2008). Since Barth (1969) seminal work, the concept of boundaries has been a key conceptual tool in ethnic studies (see Lamont and Molnár 2002; Jenkins 2008; Fenton 2010; Brubaker 2014, etc.). This is because, on the one hand, its ‘spatial and physical overtones’ (Jenkins 2008, 21) provides a concrete tool to analyse ethnicization processes (Lamont and Molnár 2002; Lamont et al. 2015) ‐ which are often related to the formation, maintenance and dissolution of boundaries (Wimmer 2013; Brubaker 2014). On the other hand, it provides a perspective on how individuals locate their ethnic identity (Wimmer 2013, Barth 1969)—because it involves distinguishing between ‘us’ and ‘them’ (Jenkins 2008; Berreby 2008). Recent work also calls for closer attention to the mechanisms through which boundaries are not only drawn, but negotiated, blurred or reconfigured through the mobilisation and conversion of various forms of capital in transnational contexts (Erel and Ryan 2019). This article builds on such perspectives to examine how migrants' access to and use of economic, cultural and social capital influences their ability to engage in boundary work.

The question of boundaries also helps us think about the relationship between social class and ethnic solidarities. So far as the former issue is concerned, boundary theorists have generally suggested that symbolic boundaries underlie social hierarchies, justifying the unequal distribution of material and symbolic resources (Lamont 1992; Edgell et al. 2020; Lamont and Molnár 2002). From the perspective of the latter issue, meanwhile, the question is about how groups remain united to maintain common interests or face common difficulties (Okamoto and Mora 2014; Li 2020). Both points emphasize the importance of focussing on social class and material matters. The maintenance of symbolic boundaries and ethnic group solidarity often stems from or is shaped by material conditions and inequality, which are closely tied to the differential access to and distribution of resources across social classes. The present article contributes to this debate by empirically demonstrating how migrants' class positions shape both their inclusion within ethnic solidarities and their distancing from other co‐ethnic groups, thus revealing the dynamic and stratified nature of intra‐group boundaries.

Moreover, ethnic studies focus on the situational nature of ethnicity (Moerman 1965; Hall 1989; Cornell and Hartmann 2006; Nair and Thomas 2012). This is reflected in the fact that, on the one hand, some terms regarding ethnicity are ambiguous, as their meanings vary depending on the time, place, and context of discussion (Li 2020). For example, the category of Chinese may involve different interpretations by different groups (Wu and Liu 2017; Yeh 2000; Yan et al. 2019). On the other hand, ethnicity does not always have salience in social life; rather, it becomes prominent under specific social, political, or institutional conditions (Wimmer 2013; Douglass et al. 2016). This requires that we should examine when, why, and how ethnicity as a social category becomes salient in everyday life.

The difference in the salience of ethnicity as a social category has also been recently highlighted by theorists of intersectionality (Piippola et al. 2009; Atewologun 2018; Bilecen 2021). As some scholars, such as Anthias (2013), have pointed out, the social categories that intersectionality focuses on, such as class, gender, and race, should not be taken for granted in social analysis, leading to their essentialization. Recent research on intersectionality shows they have different saliency in different contexts (see Anthias and Yuval‐Davis 1983; Nash 2008; Dhamoon 2011; Juan et al. 2016; Bilecen 2021). Therefore, paying attention to the varying salience of ethnicity across different contexts not only helps to reveal the internal heterogeneity within ethnic groups, but also facilitates the exploration of how ethnicity and class—as well as other social categories—intersect in more complex ways.

Accordingly, this article maintains a focus on two issues in the conceptualization of ethnicity. First, in what circumstances and to what extent does ethnicity becomes salient, and how this is related to the class position of those involved. Second, how do immigrants in different social positions draw ethnic boundaries in different ways and from various perspectives. This may further influence the ways in which they form and sustain solidarities.

## 3 Class and Migration Capitals

In Bourdieu's social theory, ‘class’ and ‘capital’ have a close and dynamic relationship (Bourdieu 1984, 2018, 1990). By expanding the concept of ‘capital’—including a series of “transubstantiations” of economic capital such as social, cultural and symbolic capital (see Bourdieu 1985, 2018; Grenfell 2012), Bourdieu understands class as a structure of social positions shaped by the interplay of multiple forms of capital (Bourdieu 1990). The volume and composition of capital can therefore be used to analyse the relative positional relationships of actors in society (ibid). This perspective helps to emphasize the multidimensional nature of social inequality and provides a theoretical foundation for understanding how non‐economic factors—such as education, culture, and power—deepen class differences (McDonough and Abrica 2021; Wallace 2017). Drawing on Bourdieu's class theory, this paper uses class to describe the relative position of immigrants in society, measured by the amount and forms of capital they possess.

Bourdieu's theories of class and capital have been widely adopted and applied in migration studies (Kim 2018; Yasin 2020). One of the most notable contributions is Erel's (2010) theory of ‘migrant capital’, which describes how migrants mobilize, transform, and create social capital within and across different social fields. Research emphasizes that migrants' ability to access and utilize various forms of capital is influenced by intersecting social positions, including gender, class, and race (Erel and Ryan 2019; Erel et al. 2016). Erel's work challenges the notion that migrant integration and capital accumulation follow a linear trajectory, revealing the complex relationship between migrants' capital accumulation and their social positioning. Moreover, Erel and Ryan's recent multi‐level spatio‐temporal analytical framework (2019) highlights the importance of accounting for diverse and shifting social contexts, further uncovering the complexities of capital formation among migrants as both ethnic and spatially mobile subjects.

Erel's research offers significant insights into the complexity and heterogeneity of migrants. The diverse ways and strategies through which migrants accumulate capital across national borders not only indicate that a given group may be divided along class and social status lines, but more importantly, they also imply that more complex forms of heterogeneity may emerge within migrant groups along other social categorizations which are often confused or overlooked. Recent studies have also noticed this issue. For example, Türkmen (2024), through a critique of ‘categorical astigmatism’, highlights how migrants are frequently reduced to specific ethnic or religious categories—for instance, the conflation of ‘Muslims’ with ‘Turks’ and ‘migrants’—thereby obscuring the significance of internal differences. This reminds us of the need to further unpack a particular immigrant group and focus on its internal heterogeneity.

This paper, therefore, holds significant implications for revealing the heterogeneity within British Chinese migrant groups. Chinese migrants are often perceived as a homogenous group (Yeh 2014) and conflated with the broader category of ‘pan‐Chinese’ (Yan et al. 2019). Some existing studies have explored the heterogeneity of Chinese identity from cultural (Yeh 2000), political (Li 2020), and class (Yan et al. 2019) perspectives. However, more research is needed to uncover the diverse experiences of Chinese migrants across different national contexts.

## 4 Citizenship and Work

The paper explores these issues in respect of two main fields that are closely related to the lives of immigrants: citizenship and work. Citizenship holds significant research value in exploring immigrant identity and experience (Doçi et al. 2024). On one hand, it represents the rights granted by the state to immigrants to legally reside, work, and participate in political and social life (Okoth 2017). On the other hand, it involves the drawing of boundaries and the construction of a sense of belonging, as it reflects how state and actors distinguish between ‘us’ and ‘them’ (Aptekar 2016).

Work is also one of the key concerns in migration studies, directly linked to immigrants' economic integration, social status, and quality of life (Painter II 2015). Occupations thus have often been used as proxies for social class. However, the link between occupation and class is complex. Savage (2015) argue that occupational positions alone cannot fully capture class dynamics, which are shaped by the interplay of economic and cultural capital, as well as lifestyle patterns. Similarly, Jonsson et al. (2009) highlight the concept of ‘microclass’ to reveal the nuanced distinctions within occupational groupings and their varying implications for life chances and social mobility. These perspectives suggest that while occupation alone cannot substitute for class, it nevertheless offers a meaningful analytical lens for exploring class‐related processes. Accordingly, this paper treats occupation not as a definitive indicator of class, but as an entry point to observe how migrants' class positions are shaped, negotiated, and manifested—particularly as they intersect with ethnicity in the domain of work. This approach is especially useful in qualitative research, where occupation can serve as a practical marker to explore broader class dynamics in migrants' lived experiences.

On the other hand, work can be nested into a broader socioeconomic context. In the face of the long‐term economic depression in the UK (Harvey 2021; Smith 2022; Zhu 2024), immigrants' strategies for work represents their ability to cope with risks, which depends largely on their social class. Moreover, analysing how specific macro‐sociopolitical contexts (Elder 1998) shape migrants' work strategies can also help to study the temporal dimension of migration mobility (Erel and Ryan 2019), which in turn helps to fill the gap in migration research in terms of understanding time and applying longitudinal research (Robertson and Ho 2016; Griffiths et al. 2013).

In the design, analysis, and presentation of data, this paper draws on a multi‐level analytical framework (Erel and Ryan 2019) to examine how migrant capital and identity are shaped across different dimensions. Specifically, at the micro‐level, it explores individual narratives and the ways migrants make sense of their experiences. The meso‐level focuses on interpersonal relationships and institutional networks, such as workplace dynamics or religious communities. The macro‐level refers to the broader structural forces—immigration policies and labour market regimes—that constrain or enable migrant agency. These levels are not treated in isolation but as interacting layers that shape migrants' lived realities and perceptions of ethnicity and class. This study thus employs life history interviews for data collection, as described in the following sections, because this approach can reveal the dynamic interplay between individuals' mobilizations and pursuit of capital and the structural forces shaped by state and societal conditions.

## 5 Methods

This study employs qualitative analysis to deeply explore the processes of ethnic identity construction and negotiation among immigrants of different classes. A purposive sampling strategy was adopted to recruit and interview 28 Chinese immigrants in the UK. The criteria for immigrants were defined as first‐generation immigrants, that is, individuals living in the host country but born in a foreign country (Carliner 1980; Borjas 1992). In order to ensure sufficient life experience as an immigrant in the UK, participants were required to have lived in the UK for at least 5 years.

### 5.1 Sampling Strategy and Class Composition

Given the focus on class and identity, ensuring a diverse class representation within the sample was critical. To this end, participants were selected to represent varied occupational, educational, and income backgrounds, as well as a balance of age and gender. In classifying participants into different social classes, this study did not rely solely on occupation as a categorical variable, but followed a multidimensional understanding of class (Savage 2015; Jonsson et al. 2009). While occupational type provided an initial entry point, the classification was further informed by indicators of economic capital, cultural capital, and social capital (Bourdieu 1984). Economic capital included income and job security; cultural capital encompassed educational level, linguistic repertoire, and familiarity with institutional contexts; and social capital referred to professional and community networks.

Therefore, participants classified as middle class tended to hold university or postgraduate degrees and stable professional occupations (e.g., lecturers, lawyers, engineers), displaying relative ease in navigating formal institutions. Those categorized as working class typically engaged in manual or service‐sector work (e.g., restaurant staff, delivery drivers, barbers), reported greater employment precarity, and expressed more distance from bureaucratic and academic settings. Moreover, participants' self‐perceptions and narratives of mobility were also considered, reflecting a relational understanding of class as a lived and negotiated identity (Savage 2015).

### 5.2 Fieldwork and Interviews

The recruitment of participants was divided into two stages. The initial outreach relied on personal referrals, followed by snowball sampling. However, in view of the selection bias and homogenization risks of snowballing (Parker et al. 2019), the class and ethnic experiences of social actors who are in different positions in the same power structure second stage strategically targeted migrants across multiple social spaces and diverse social positions. This not only increased the heterogeneity of the participants, but also facilitated the acquisition of data on the and have daily interactions (Grenfell 2012). The final cohort of 28 participants represented a wide range of occupations spanning both working‐class and middle‐class positions. These included university lecturers, engineers, lawyers, doctors, and bank employees, as well as barbers, delivery drivers, supermarket staff, and restaurant workers. This diversity aimed to capture variation across educational attainment, income levels, and job security. Their ages ranged from 20 to 60 years old, with a gender ratio of male to female at 1:2.

After signing informed consent forms, participants were invited to be interviewed in their preferred and convenient format, including both face‐to‐face and virtual modalities. Most of them chose to conduct face‐to‐face interviews, which facilitated the observation of non‐verbal data such as gestures and micro‐expressions, which was considered important in improving the accuracy of participants' responses and improving the quality of qualitative data (Foucault Welles et al. 2022). Three participants chose virtual interviews via WeChat voice calls due to work constraints in the service industry—a choice that itself hints at class‐mediated disparities in control over leisure time and occupational flexibility (Seiluri et al. 2011).

All participants were invited to engage in life history interviews focussing on their lived experiences before and after immigrating to the UK. This methodological choice serves a triple purpose. First, life history interviews generate cohesive narratives about individuals' trajectories, experiences, and affective dimensions across distinct life stages. Such narratives not only facilitate the capture of evolving understandings of identity over time but also illuminate the longitudinal interplay between class dynamics and ethnic identity formation. Second, and more importantly, they unveil the ‘tangle of relationships and symbiotic interactions’ (Jessee and Hinch 2019, 2, see also Jackson and Russell 2010) between personal memory and those that circulate in the broader sociocultural frameworks in which the individual is embedded, thereby anchoring individuals' perceptions of ethnic belonging to their unique biographical trajectories. Therefore, as Erel and Ryan (2019) emphasize, focussing on participants' micro biographies can embed individual lives into a larger macro context and help us to think about the relationship between ethnicity and class from a longitudinal perspective. Additionally, this approach decentralizes researcher authority by positioning participants as narrative agents, mitigating power asymmetries inherent in interviewer‐interviewee dynamics (Jackson and Russell 2010, 8).

Considering that class is not something ordinary people typically reflect on explicitly, the interviews were carefully designed to evoke reflections on the longitudinal interplay between class dynamics and ethnic identity. To achieve this, participants were first invited to share personal stories about their everyday experiences. Follow‐up questions then probed deeper into how their backgrounds shaped opportunities and constraints over time. To guide these conversations, open‐ended prompts such as: ‘How did your work and life change after moving to the UK?’, and ‘Have you ever felt different from others here?’ were used. These questions were designed to surface implicit understandings of class positioning and social comparison without relying on abstract or academic terminology. This approach embedded class within everyday narratives, allowing participants to express classed experiences through stories of work, family roles, and social belonging—thereby capturing more effectively the evolving relationship between class and ethnic identity over time.

### 5.3 Language and Translation

Interviews were conducted in participants' preferred language, including Mandarin and English. Interviews generally lasted 3–4 h. Some participants were interviewed multiple times, with the longest interview lasting up to 8 h in total. All interviews were audio‐recorded for subsequent analysis. All recordings were transcribed after the interviews and translated into English for further analysis when necessary. Acknowledging the inevitable challenges in cross‐cultural translation, particular care was taken throughout the process. Drawing on the principles of culturally responsive translation (Temple and Young 2004), special attention was given to preserving culturally embedded meanings through strategies of equivalence and difference during both transcription and translation. To this end, bilingual researchers were invited to review the transcripts for semantic accuracy, and key expressions were cross‐checked against the original audio to minimize the loss of subtle nuances. For concepts that lacked direct equivalence in English—raising issues of translatability—contextual annotations were added to maintain interpretive clarity. Anonymization and blurring of potentially identifying details were performed to protect participants.

### 5.4 Data Analysis

Given the study's exploratory aims, thematic analysis was adopted. The analysis process was broadly divided into three steps. First, the transcripts were reread and initially coded. Codes related to migrant identity were identified through manual coding, and organized into thematic clusters. These clusters were contextualized within participants' life stories to isolate class‐inflected statements, which were systematized into patterns shaping identity articulation. Finally, cross‐case comparisons of identity narratives and social positioning revealed divergent pathways through which class mediates ethnic identity.

These analytic steps were embedded in a broader interpretivist and constructivist epistemology, which views class and ethnic identity not as fixed categories but as contextually constructed through lived experience and social interaction. Given that participants may not consciously articulate their class or ethnic position, meaning‐making occurs through how they describe work, belonging, and life transitions. As a researcher, I took an active interpretive role in identifying how these narratives reflect underlying social structures and evolving identities. Drawing on a phenomenological sensitivity, the analysis prioritises participants' own perspectives, while also acknowledging the researcher's influence in interpreting patterns across stories. Reflexive memos were used throughout the analysis process to document and critically assess my positionality, assumptions, and meaning‐making frameworks.

### 5.5 Reflection and Limitations

Despite efforts to ensure occupational and class diversity within the sample, several limitations of the methods should be acknowledged and critically reflected upon. First, although the research includes participants from both working‐class and middle‐class backgrounds, it does not capture the perspectives of highly affluent or elite migrants. This omission may limit a fuller understanding of how privilege operates within migrant communities and may unintentionally amplify narratives of class disadvantage. Second, although purposive and snowball sampling facilitated access to a range of participants, it also introduced potential clustering effects. In particular, a number of participants were recruited through Chinese church networks in Scotland, which may have led to the overrepresentation of certain community values, such as religious solidarity, while underrepresenting secular or individualized adaptation strategies.

Finally, although interviews were conducted in participants' preferred languages to ensure linguistic ease and comfort, this inevitably influenced the manner, nature and depth of disclosure—especially on sensitive topics such as ethnic discrimination, belonging or identity. Furthermore, my own positionality as a bilingual researcher with a Chinese background, while enabling cultural proximity and rapport, may have consciously or unconsciously shaped participants' narratives—as the interview is a co‐constructed process in which the researcher and the participant jointly construct knowledge through interaction and dialog (Larkin et al. 2007). These dynamics were considered through reflexive journaling and critical memoing during the research process, but they nonetheless shape the interpretive lens through which the data were collected and analysed.

## 6 Findings

When the participants talked about their experiences before and after moving to the UK, almost all of them reiterated the numerous difficulties they faced during their migration and their different coping strategies. The barriers involved in crossing national borders and how they dynamically mobilized capitals in the course of navigating those barriers, were strongly reflected (Erel and Ryan 2019). What is worth noting here is that the different attitudes they articulated when discussing these barriers, such as resignation, helplessness, or indifference, were often elevated into ethnic stereotypes and influenced the drawing of symbolic boundaries.

These accounts suggest that perceptions of ethnicity may be shaped not only by cultural background but also by structural experiences in the migration process. As participants described their interactions with institutions, legal systems, and everyday encounters, their sense of ethnic belonging appeared to emerge in specific contexts—particularly in moments of exclusion or marginalization. While participants differed in their backgrounds and life trajectories, the interviews revealed similarities in how individuals reflected on ethnicity and belonging across social positions.

The following sections explore these themes in greater depth by focussing on two major domains central to immigrant life—citizenship and work—in order to understand how social class intersects with ethnic identity in shaping migrant experiences.

### 6.1 Citizenship: Transnational Barriers and Ethnicity Salience

Proving one's right to reside and work in the destination country is a fundamental and immediate challenge for every immigrant. For Chinese immigrants in the UK, this is manifested through the need to obtain visas and provide legal documentation of residence and a right to employment for employers.

The process of obtaining legal status is often more difficult and tortuous for participants in lower classes. Paul, who works as a barber in the UK, complained in an interview about the high visa and lawyer fees. ‘The visa fee was too expensive… They're robbers.’ Facing the visa lawyer recommended by his friend, he cursed: ‘They charge by the hour—£150. That's daylight robbery!’ he complained to me. ‘Just applying for a visa would cost me months of income. I'd just come here to open a shop—how could I afford that?’ The visa application, which had been submitted with great difficulty, was soon followed by a long period of waiting. He was eager to get the visa approved as soon as possible because it affected his capacity to work and his rent—unscrupulous landlords were able to charge him more because he was vulnerable due to identity issues. Although he knew there was options to fast‐track process, the high cost discouraged him. All of this made him extremely anxious. When recalling these events, he spoke with deep indignation: ‘British devils are inefficient… They're lazy and incompetent’.

Paul's experience highlights the prolonged torment that comes with waiting (Stewart and Sanders 2024). Ethnicity becomes particularly salient in this process, as reflected in Paul's angry remarks and the resulting stereotype he developed about the British—inefficient, devils, robber. This reinforces his sense of ethnic boundaries and adds to the way he understands ethnicity. Paul thus saw himself as an ‘outsider’, and conveyed a sense of being excluded: ‘I don't belong here. They don't treat me as one of them’.

Several participants shared experiences similar to Paul's. Supermarket worker Xena, restaurant owner Wesli and chef Kayli all voiced similar frustrations about the difficulties of obtaining legal status in the UK. Their complaints eventually turned into expressions of dissatisfaction towards British people, whom they described as ‘incompetent’ (Xena), ‘lazy’ (Wesli), and ‘lacking humanity’ (Kayli). These experiences of waiting, exclusion and frustration not only shaped participants' perceptions of British society but also influenced how they understood themselves. For those from lower social classes, the long and expensive visa process became a moment in which both class and ethnic boundaries were sharply felt. Paul's sense of being an ‘outsider’, for instance, stemmed not only from his perception of the immigration system as inefficient, but also from his inability to access the services that wealthier migrants could afford. This sense of exclusion, made visible through contrast, was experienced at the intersection of class and ethnicity. The derogatory terms used by Paul and others—such as ‘lazy’, ‘devils’, ‘lacking humanity’—can be understood as a reactive form of boundary‐making that reflected and reinforced their sense of marginalization.

These narratives show that the difficulties in securing legal status did not merely cause personal stress but prompted them to reinterpret their social positioning in the UK—as migrants who are simultaneously disadvantaged by class and racialized through perceived systemic neglect. These experiences deepen their understanding of themselves as members of a marginalized ethnic group, shaped by broader structures of inequality. *These visa struggles, therefore, not only reflect micro‐level anxieties about belonging but are also deeply embedded in macro‐level immigration policies that institutionalize unequal access to legal protections. Moreover, interactions with landlords and visa agents reveal meso‐level power dynamics, which mediate how migrants interpret exclusion.* Moreover, such attitudes were not formed immediately, but became increasingly entrenched through repeated experiences of exclusion. These experiences underscore a temporal dynamic—participants’ perceptions of marginalisation often intensified with longer residence in the UK. What initially seemed like bureaucratic inefficiencies were later interpreted as classed or racialised disadvantages. This illustrates that the salience of ethnicity in migration is not fixed, but shaped by cumulative encounters with structural barriers over time.

In contrast, participants from higher classes often faced fewer obstacles and handled them more easily. Xerxes was born in a more affluent family. He chose to stay in the UK and start a business after graduating with an MBA from an England university. When he was interviewed, he was running a cross‐border e‐commerce company. When talking about his experiences related to proving his legal status in the UK, he mentioned that his first student visa application was ‘indeed a bit troublesome, with many options and the requirement to submit documents at designated locations’. Therefore, he decided to hand the process over to an agency and opted for Priority service. ‘Why? You could have just waited a few extra days and saved money’, I asked. He shook his head and replied, ‘Let me tell you, if a problem can be solved with money, it's not a real problem’. Xerxes' philosophy is to ‘spend money to buy time’. He believes that if money can make things happen faster, there is no need to wait. Therefore, for him, the expedited service provided by the UK immigration authorities was a sign of friendliness and professionalism. As for visa issues after he started working, he did not mention any in his narrative. When I asked him about it after his personal account concluded, he said, “Oh, I don't really know much about that—my assistant handled it for me.” By stark contrast with Paul, Xerxes said he did not feel much exclusion in the UK and viewed the Priority visa service as ‘quite humane’. Moreover, he saw such policies as a reflection of British inclusiveness, as they consider various needs and provide thoughtful solutions. Ethnicity is diluted in the process, which facilitates his integration. ‘At least from the British people I've come into contact with, I feel that they're all friendly and inclusive’ he said to me.

These cases highlight the critical role of class in both crossing national boundaries but also how, in turn, it informs the construction of ethnic identity. Immigrants from different class backgrounds face varying degrees of obstacles when attempting to prove their legal status, and this largely depends on the capital they possess or can mobilize, as well as the services they can afford. Xerxes could afford Priority services, which allowed him to reduce waiting time. Furthermore, he can entrust the visa application process to intermediaries and assistants, thereby getting rid of the trouble of the process, which also reflects his ability to mobilize capital. Obtaining legal status thus never seemed like a significant barrier to him; instead, the ability to pay more for more convenient services was perceived as a sign of humanized, considerate treatment.

In contrast, participants at the lower level, like Paul could not afford legal assistance, and had to face the visa process alone. The language barrier added to his difficulties and anxiety. Additionally, because he could not afford what was, for him, the high cost of Priority services, he was forced to endure a long wait even though he was in urgent need of a visa. For Paul, this experience of waiting was a prolonged suffering (Stewart and Sanders 2024), as he was unable to control the present or predict the future (Bourdieu 2000). The Priority service of visa should be seen as a reflection of class inequality, as it relies on paying extra fees for faster service and additional convenience, which inherently favours the wealthy and places the poor at a disadvantage. Immigrants or social classes unable to afford these costs may thus face institutionalized injustice (Czaika et al. 2018), reinforcing class‐based disparities (Chung 2020). From this perspective, the hierarchies migrants occupy in their country of origin are not dissolved upon migration, but may be reproduced—or even exacerbated—in the host society. Participants from middle‐class backgrounds, such as those with elite university degrees or urban upbringings, often leveraged these pre‐existing advantages to navigate complex immigration procedures more efficiently. In contrast, those from rural or working‐class backgrounds lacked the resources or institutional familiarity to do the same. As Trinidad and Faas (2025) argue, economic and cultural capital acquired in the country of origin does not vanish at the border but is revalued and restructured in transnational contexts—contributing to the reproduction of familiar social hierarchies abroad.

It is worth noting that in this process, gender and visa status also intersected to shape participants' experiences of exclusion. For instance, several female participants who came to the UK on spousal or dependent visas expressed greater vulnerability, as their legal and financial status was tied to their partners. This legal dependency often restricted their access to work and intensified feelings of marginalization. By contrast, those who entered the UK via student or skilled worker visas—predominantly middle‐class participants—navigated the immigration system with more autonomy and institutional familiarity. These intersecting factors further complicated how participants made sense of their social positioning in the UK.

This series of class‐based experiences further influenced their attitudes towards British society and their understanding of ethnicity. On one hand, participants from lower class, like Paul, were tormented by the limitations and difficulties they faced during the waiting period for legal status. The lack of legal status limits their participation in social and political activities—in fact, more restrictions on basic life, such as accommodation—which magnified the boundaries and led to a deep sense of exclusion. Ethnicity became highly salient during this process. However, it is important to note that this salience was often triggered by class‐related vulnerabilities. The exclusion these participants experienced was not purely a matter of ethnicity; rather, it was shaped by limited access to cultural capital—such as low English proficiency, unfamiliarity with legal procedures, and minimal institutional knowledge. These disadvantages—deeply rooted in class position—intensified their sense of marginality and led them to interpret their experiences through the lens of ethnic identity. In this sense, it was not ethnicity per se that produced exclusion, but rather the material and symbolic constraints associated with class. Ethnicity was made visible, or felt more acutely, because class‐based disadvantages shaped how participants navigated legal systems and social structures. The experience of being ‘Chinese’ in the UK was thus filtered through a classed reality that determined what kind of British society one could access.

Furthermore, poorer migrants find themselves placed in an antagonistic relationship with the immigration services during crossing bordars. It is within the structure of that antagonistic relation that ethnicity intervenes and becomes a salient factor, a way of making sense of what is happening. On the other hand, the very same procedures that caused Paul so much suffering were seen by participants like Xerxes as commendable aspects of the UK, because he can enjoy more convenient services by paying higher fees. In other words, for participants in higher classes, their wealth allows them to avoid that antagonism. Therefore, ethnicity was not so salient as a mode of explanation of their lived experience of those relations.

Moreover, these contrasting experiences reveal more than just personal struggles—they point to deeper structural dynamics that shape migrants' life chances. As Weiss (2005) argues, transnational social inequality is reproduced through state policies that favour migrants with specific forms of cultural and economic capital. The UK immigration system, for instance, offers expedited services for those who can pay more, thereby institutionalizing advantages for wealthier migrants while systematically disadvantaging those from lower‐class backgrounds. Migrants like Paul, Xena, and Kayli face compounded difficulties not only because of personal resource limitations, but also because immigration regimes are designed in ways that align with the needs of the host country's labour market and economic goals. These policies reflect a broader class‐based logic that rewards those who can demonstrate ‘desirable’ skills, credentials, or capital, and marginalizes those who cannot. Viewed from this perspective, the struggles lower‐class migrants face in securing legal status are not merely bureaucratic challenges but manifestations of transnational inequalities embedded in state frameworks.

This also suggests that the exploration of identity should always be conducted through multiple levels and dimensions—just as Erel and Ryan's (2019) multi‐level analytical framework advocates. More specifically, individuals' understandings of ethnicity are shaped not only by personal feelings of exclusion (as in Paul's case) or inclusion (as in Xerxes'), but are also deeply connected to interpersonal power dynamics (such as exploitation by landlords or visa agents), as well as macro‐level state structures (such as visa regimes).

### 6.2 Work Strategies: Migrant Capitals and Ethnic Boundaries

Many participants used career development as a narrative thread, making ‘work’ a particularly salient theme in the data analysis. As established in existing scholarship, work not only underpins migrants' economic integration but also plays a crucial role in shaping their social positioning and identity formation. In this section, I argue that migrants' work strategies influence how they draw ethnic boundaries and build the forms of ethnic solidarity.

#### 6.2.1 Boundary Delineation

Most participants mentioned the increasingly challenging employment environment in the UK over the past decade, including more stringent entry requirements and a continual reduction in available job vacancies. They also expressed concerns about the resulting rise in living costs, fears of layoffs, and the growing difficulty of (re)entering the workforce. This is supported by research, which highlights the UK's ongoing economic downturn in recent years (Harvey 2021) and its wide‐ranging effects—such as increased income inequality across regions in the UK (Smith 2022), as well as impacts on the labour market (Zhu 2024), particularly the rising unemployment rates among ethnic minority groups (e.g., Šestanović et al. 2021). The analysis shows that immigrants from different class backgrounds respond to social change with varying attitudes and strategies.

For example, Zheng, a waiter at a Chinese restaurant, mentioned the rapid rise in living costs after the pandemic and the immense financial pressure he has had to bear. ‘Prices are going up; rent is going up too. Just look at housing prices, I don't even know where I can live anymore’. The resulting instability in his work situation had also been a source of distress.

The economy is bad. My previous (Chinese restaurant) was about to shut down, so they even stopped letting us eat there… I had to move to this current restaurant, but business here is also getting worse and worse. I don’t know how much longer I can keep this job.

Due to his limited education and lack of English skills, his job options in the UK were restricted. “If I'm in China, I could still look for other kinds of work, but here, this is all I can do, because I don't speak English.” By chance, he was introduced to a Chinese church through a friend. ‘There are many Chinese people from different (professions) in the church, like a lot of restaurant owners, so I can quickly find a job if I ever (lose one)’. This gave him a sense of security and belonging. ‘Joining the church was really good. Everyone helps each other, and I feel like only Chinese people can truly look out for each other’.

Zheng's story illustrates that immigrants in lower class often bear more negative consequences in the face of social transformations due to their limited ability to cope with risks. This vulnerability is closely tied to the scarcity of resources they possess and their constrained ability to convert capital—such as structural barriers caused by language barriers and low educational attainment, which further restrict their occupational choices. As the economy declined, Zheng faced increasing difficulty in meeting basic survival needs like food and housing. This situation exemplifies what Bourdieu (1990) refers to as the ‘hysteresis effect’—a mismatch between one's ingrained dispositions (or habitus) and a rapidly changing social environment. In other words, when the social field changes faster than individuals can adapt, their existing dispositions become maladaptive. Zheng's class‐based habitus, shaped by his past experiences and available capital, left him ill‐equipped to respond to the evolving structure of British society. In such moments of dislocation, ethnicity became particularly salient, as his social positioning reinforced his sense of difference and exclusion. Meanwhile, the work and life strategies adopted by him and others in similar social positions—based on the limited capital they can utilize and access, such as networks of friends in comparable circumstances—led to a greater reliance on the Chinese community and internal resources. This reinforced the maintenance of ethnic boundaries. Therefore, Zheng's experience is shaped not only by his individual constraints (micro‐level) but also by the lack of institutional support and segmented labour market (macro‐level), as well as the informal employment networks that circulate within Chinese communities (meso‐level). These overlapping factors co‐produce a particular classed and ethnicized experience of work.

This is also reflected in the experiences of other Chinese immigrants I interviewed at the same church. Chen, a chef at a Chinese restaurant, had worked at four different restaurants before settling at his current job due to poor business conditions. The instability brought on by frequent job changes left him physically and emotionally exhausted, leading to a shift in his attitude towards the UK. ‘I feel like coming to the UK was a mistake. I used to think about bringing my family over, but now I don't think that at all. I've realized that we can never truly belong here’. However, returning to China was not an option he could accept, as he saw it as a form of failure—just as many immigrants do (Sayad 2018). In a moment of despair, he joined the church through a friend's introduction. ‘Great, because I've received a lot of help here. They've helped me get through many (difficult times)’.

By contrast, participants from higher social class often demonstrated greater ease and flexibility in coping with challenges. For example, Ella, who holds a PhD, was working as a research assistant at a university in the UK at the time of the interview. Although she also felt the pressure of rising living costs — ‘I've now moved to XXX [a relatively remote area compared to where she lived before]. The rent for my previous place went up by £35 per week. Things've gotten ridiculously expensive after the pandemic’—the effect on her life was relatively limited. For instance, she cancelled a planned trip to France 2 months later, because ‘that damn moving company cost me a lot’.

The economic downturn and reduction in job opportunities also influenced her career strategies. ‘Two months ago, a good university in London gave me an offer—the salary was a bit higher than here’, she said, ‘but the high cost of living in London made me give up on it’. Fortunately, despite her university announcing plans to lay off more than 1000 staff members this year, she felt secure in her position: ‘Our research team needs staff who can speak Mandarin, so both I and another Chinese lady in the department don't need to worry’. Her experience in the UK has fostered a strong sense of belonging to British society. In her account, British people are described as ‘more friendly’ and ‘more egalitarian’.

These perceptions have made ethnic boundaries appear more blurred and fluid for Ella, which is reflected in the way these boundaries are drawn differently across various contexts in her account of her experiences. For instance, in most every day and workplace scenarios, she finds certain traits of the British people she interacts with more comfortable compared to her impression of Chinese people. She was thus more inclined to identify with the British as ‘us’*—suggesting a tendency to position herself as a cosmopolitan subject in that context*. However, when she mentioned that the team requires Mandarin‐speaking staff, or when colleagues seek her input on Mandarin‐related texts or issues, her Chinese identity becomes foregrounded. Furthermore, this flexibility, from another perspective, also unfolded temporally. For example, Ella initially expressed a strong sense of professional belonging and limited ethnic salience. However, over time, certain situations—such as being recognized for her ability to speak Mandarin or encountering subtle forms of exclusion and limitation in her career development—prompted her to occasionally re‐identify with her ethnic group. This indicates how prolonged exposure to local institutions can reshape previously ‘cosmopolitan’ identity narratives.

Unlike Zheng and Chen, Ella, positioned in a higher social class, possesses more capital—such as a doctoral degree and proficient English skills. These enable her to access a wider range of higher‐paying job opportunities, freeing her from concerns over basic survival needs like food and housing. At the same time, her existing resource—such as Mandarin language proficiency—can be converted into locally valuable professional capital in the UK, allowing her to build closer connections with mainstream society. On the contrary, for Zheng and Chen, this very aspect becomes a barrier to integration. Therefore, while working‐class immigrants tend to reliant on forms of support and social capital available ‘within’ the boundaries of ethnic communities, Ella's ability to navigate and redefine boundaries through her class advantages has shaped a more fluid and hybrid ethnic identity. This contrast reflects not only present class status but also the long‐term accumulation of capital from the country of origin. For instance, Zheng's limited mobility partly stemmed from his rural background and lack of institutional exposure in China—disadvantages that continued to constrain him in the UK labour market. Conversely, Ella's urban origin and professional background gave her translatable advantages. These examples support Trinidad and Faas's (2025) observation that economic and cultural capital acquired pre‐migration is often re‐activated or re‐stratified in the host society.

Moreover, regional origin emerged as a notable factor that often intersected with class to shape participants' migration trajectories. Participants from urban backgrounds—such as Ella, who grew up in a big city in northern China—typically had access to more formal education and familial resources, enabling them to enter white‐collar professions more easily. In contrast, those from rural areas—like Zheng, who came from a village in southeastern China—were more likely to work in the service sector, often due to limited family support and fewer educational opportunities. These unequal distributions of resources, rooted in regional disparities, had significant implications for migrants' paths in the UK. These regional disparities—carried across borders—interacted with class and influenced how participants navigated the UK labour market and understood their migrant status.

These cases also highlight how class habitus operates across multiple levels to shape the delineation of ethnic boundaries: at the individual level, through migrants' dispositions and coping strategies—for example, Zheng's precarious situation versus Ella's flexibility; at the interpersonal level, through workplace interactions and negotiations; and at the structural level, through labour market segmentation and rising living costs. These layers are intersected, jointly influencing how participants in different social positions perceive and construct ethnic boundaries in divergent ways.

#### 6.2.2 Ethnic Solidarity

Work strategies therefore highlight a wider point about how migrants of different social classes form distinct types of solidarity and how the process shape their ethnic identities. Ethnic solidarity is examined here because it serves both as a strategy adopted by migrants and as a factor that shapes their ethnic identity. As some sociologists have pointed out, ethnic solidarity and ethnic identity are not always the same thing (Okamoto and Mora 2014; Li 2020). The former refers to one identifying as part of an ethnic group based on shared cultural traits, while the latter emphasizes individuals from different backgrounds uniting on the basis of shared experience, language or cultural (Rosenfeld 2001).

For participants from lower social classes, such as Zheng and Chen, the capital they possess and can mobilize is limited—they lack economic resources, do not speak English, and have few connections outside their ethnic group. As a result, they tend to join co‐ethnic communities to collectively cope with changing social conditions. It turns out that joining Chinese churches not only expanded their social networks but also provided access to shared material resources and job opportunities, placing them in a more advantageous position during economic downturns compared to facing challenges alone. This also strengthened ethnic boundaries and deepened their awareness of ethnic identity ‐ for them, this only underlined their sense of not belonging to the British group. This is reflected in Chen's words,

After coming to the church, I feel that life is a little bit easier, find jobs, make friends … It's hard for Chinese people to live here if they don't stick together. British don't care about you. They don't consider us as their own people.

Similar experiences were also shared by driver Richard. Richard spoke in the interview about joining an online group for Chinese tour drivers in the UK, excitedly noting that this connection had brought him 35% more bookings than usual. ‘This is great. I've finally found one of my own’. He said to me.

In contrast, participants from higher social classes, equipped with greater capital—especially social and cultural capital—had more options in forming social networks and modes of solidarity. For example, Ella could comfortably and flexibly engage with people from various ethnic backgrounds, and align herself with different groups in different contexts, resulting in a more fluid and hybrid sense of ethnic identity. For instance, she aligned herself with British people when discussing welfare and education but identified as Chinese when talking about politics and culture.

These experiences reveal the differing forms of solidarity among migrants from different social classes, and how such solidarities contribute to the construction of ethnic boundaries. The result shows that, on the one hand, the solidarity formed by lower‐class participants does not always align with a broad ‘Chinese’ identity, it often follows different trajectories—such as shared faith, occupation, or regional origin. Despite this, however, due to their limited capital and lack of ability to convert it, these participants tend to form alliances with co‐ethnic groups in similar social positions. This is because they face similar difficulties and this strategy allows them to not only pool resources and better respond to social changes, but also to maximize the value of their distinct forms of capital. Such alliances reinforced the boundary between their respective communities and the British majority. In contrast, participants from higher social classes tend to exhibit more flexible forms of solidarity and construct more dynamic and diverse ethnic boundaries.

The formation of solidarity patterns also reflected longitudinal shifts. Among working‐class participants, prolonged instability in the labour market often intensified reliance on ethnic networks over time, leading to more rigid boundary‐making. What began as pragmatic job‐seeking strategies gradually developed into stronger ethnic identification, particularly when systemic exclusion persisted. Conversely, for some higher‐class migrants, sustained integration and cultural capital occasionally led to weakened ethnic ties, though this was not always linear. Furthermore, this also suggests that the patterns of solidarity observed among participants were also shaped by their pre‐migration social positioning. Middle‐class individuals, equipped with more varied forms of capital, were often able to maintain connections across different groups, thereby achieving a certain degree of social distinction; in contrast, working‐class migrants tended to engage primarily with others of similar class and regional background due to their limited resources (Bourdieu 2018; Zhang 2010). This reflects deeply ingrained hierarchical perceptions rooted in Chinese society, which subtly shaped whom they interacted with and how. These divergent tendencies highlight how classed hierarchies originating in China continued to shape migrants' network boundaries and solidarity practices in the UK context, reinforcing the transnational continuity of class‐based divisions (Trinidad and Faas 2025).

Moreover, these variations in solidarity practices further reflect the multi‐level nature of identity construction (Erel and Ryan 2019). On one hand, shared structural constraints—such as limited job opportunities or housing discrimination—shape interactive support patterns, such as church networks. On the other hand, these support patterns in turn reinforce individuals' perceptions of ethnic belonging. In other words, ethnic identity construction thus emerges from the interplay between macro‐level structural limitations, meso‐level community‐based mechanisms, and micro‐level individual trajectories of belonging.

## 7 Conclusion and Discussion

Based on Erel's theory of migrant capital, this article explores how migrants' different strategies and abilities to mobilize and utilize capital shape their understanding of their own ethnic identities. The findings not only validate Erel's framework but also contribute to a deeper understanding of the complexity and non‐linearity of migrant integration processes. Meanwhile, the article highlights the role of class in the construction of ethnic identity, thereby contributing to sociological discussions on the intersection of ethnicity and class. Moreover, the article reveals how Chinese migrants in the UK form solidarities along class lines, thus contributing to the exploration of heterogeneity within British Chinese migrant communities.

At a theoretical level, the article also advances boundary theory by tracing how migrants actively negotiate ethnic boundaries in relation to their access to—and conversion of—economic, social, and cultural capital. These negotiations were not fixed but context‐dependent, revealing how boundaries were reinforced, blurred, or softened depending on migrants' perceived inclusion or exclusion in different social arenas.

Focussing on two domains closely tied to migrant life—citizenship and work strategies—the findings highlight three ways in which class influences participants' experiences and perceptions of ethnicity. Firstly, class influences the extent of transnational barriers migrants face, as well as their ability to navigate these barriers through the capital they possess and can mobilize. This results in varying degrees of ethnic salience for migrants of different social class during this process. For example, lower‐class participants often cannot afford expensive visa fees and legal services, forcing them to endure long waiting periods and the associated inconveniences. This increases the difficulty of obtaining legal status, thus heightens their sense of exclusion, and fosters prejudice and stereotypes towards British people.

Secondly, class shapes migrants' future planning by influencing their ability to respond to social change and convert capital, thereby playing a role in the process of defining ‘us’ and ‘them’. The findings show that participants from lower social classes tend to turn to ethnic communities for support due to shared language and culture. This reinforces ethnic boundaries. In contrast, higher‐class participants, with stronger coping capacities, greater ability to convert capital, and more flexible employment options, tend to exhibit a more fluid, blurry and hybrid approach to ethnic boundary‐making and identity positioning. In this sense, the paper contributes to migration studies by offering an empirically grounded account of capital conversion, showing how the value of certain forms of capital—such as elite educational credentials or institutional fluency—shifts across national contexts. These shifting valuations, in turn, condition the opportunities migrants have to cross, reframe, or uphold symbolic boundaries.

Finally, this article emphasizes how class shapes the construction of different boundaries by influencing migrants' modes of ethnic solidarity. Lower class participants, due to limited capital possession and weaker capital conversion capacity, tend to unite based on the limited available capital with co‐ethnic groups because of their shared language, culture, and experience. In contrast, higher‐class participants, with access to more diverse forms of capital, are able to form more flexible alliances with groups from various backgrounds.

This article also highlights several research gaps. For example, although this article reveals that migrants of different classes tend to exhibit distinct forms of solidarity, it does not fully capture more nuanced forms of solidarity. Therefore, further research is needed to explore the more complex and detailed ways in which ethnic solidarity that unfold along class lines. Future work may also examine how boundary negotiation and capital conversion evolve over time, particularly in response to shifting macroeconomic and political structures—a point that this paper begins to address but does not exhaust.

## Funding

The author has nothing to report.

## Ethics Statement

This study has received ethical approval from the University of Glasgow Ethics Committee.

## Consent

Informed consent was obtained from all participants involved in the study.

## Conflicts of Interest

The author declares no conflicts of interest.

## Data Availability

Research data are not shared.
